# In vivo mapping of protein-protein interactions of schizophrenia risk factors generates an interconnected disease network

**DOI:** 10.1038/s41537-026-00734-1

**Published:** 2026-03-07

**Authors:** Daniel B. McClatchy, Jeff Lane, Susan B. Powell, John R. Yates III

**Affiliations:** 1 Integrative Structural and Computational Biology Department, The Scripps Research Institute, La Jolla, CA USA; 2 Department of Psychiatry, UCSD, La Jolla, CA USA; 3 Research Service, VA San Diego Healthcare System, La Jolla, CA USA

**Keywords:** Molecular neuroscience, Cellular neuroscience, Schizophrenia

## Abstract

Genetic analyses of schizophrenia (SCZ) patients have identified thousands of risk factors. In silico protein-protein interaction (PPI) network analysis has provided strong evidence that disrupted PPI networks underlie SCZ pathogenesis. In this study, we performed in vivo PPI analysis of several SCZ risk factors (i.e., Grin2b, Grm5, Gsk3b, Map2k1, Ppp1ca, Stx1a, Syngap1, and Syt1) in the rodent brain. Using endogenous antibody immunoprecipitations analyzed by liquid chromatography coupled to mass spectrometry, we constructed a SCZ network comprising 1612 unique PPI with a 5% FDR. Over 90% of the PPIs have not been previously reported. AlphaFold3 was employed to identify direct PPI interactors. Our SCZ PPI network was enriched with known SCZ risk factors, which supports the hypothesis that an accumulation of disturbances in selected PPI networks underlies SCZ. We used Stable Isotope Labeling in Mammals (SILAM) to quantitate phencyclidine (PCP) perturbations in the SCZ network and found that PCP weakened most PPI but also led to some enhanced or new PPI. These findings demonstrate that quantifying PPI in perturbed biological states can reveal alterations to network biology.

## Introduction

Understanding how proteins interact to form networks is essential to understanding human disease pathogenesis^1^. A single genetic coding mutation can disrupt an entire protein-protein interaction (PPI) network or create a disease-specific network^2^. Since proteins form associations with multiple proteins, a single gene mutation could perturb numerous PPI networks, which may be why one mutation can cause multiple disease phenotypes. In diseases with multiple genetic causes or risk factors, gene products associated with the same or similar diseases tend to interact with each other^3,4^. It is posited that multiple genes that confer low to moderate vulnerability to a disease individually can produce more significant disease potential when they are located within the same PPI network^5^. Understanding which networks are disrupted in a disease state could reveal the mechanistic details that underlie the disease.

Analysis of PPI holds great promise for understanding the pathogenesis of schizophrenia (SCZ). SCZ is a chronic, debilitating disease which manifests as positive symptoms (i.e. hallucinations and delusions), negative symptoms (i.e. emotional withdrawal and social interaction deficits) and cognitive dysfunction^6^. SCZ patients have difficulty successfully integrating into society, an increased risk of suicide, and a reduction in life expectancy^7,8^. It is estimated that the prevalence of SCZ in most populations is 1%^9^. Drug treatment of SCZ has not significantly improved since the development of antipsychotics in the 1950s, and antipsychotics only alleviate positive symptoms. The dearth of pharmacological treatments highlights the lack of mechanistic understanding of the pathogenesis^10^. SCZ is a disease of high heritability. The complex polygenicity among different populations, combined with the involvement of environmental factors, has made finding drug treatments and cures challenging. Of the ~6000 genes that have been linked to SCZ pathogenesis, only ~9% have been assigned a causal relationship^11^. There are a few rare genetic risk factors that individually confer a relatively high risk, whereas more common risk factors are postulated to act in an additive manner to trigger disease^12^. PPI disease network analyses have demonstrated that SCZ risk genes interact more significantly with other SCZ risk genes than with genes with no SCZ association^13–15^. These analyses have identified previously unknown risk factors that are integral components of enriched SCZ-relevant pathways^11,15^. There is great optimism for future treatments now that in silico PPI analyses have begun to unravel the complex genetic landscape of SCZ.

In silico PPI analysis is hindered by the need for a priori knowledge of a gene’s function^16,17^. Tissue-specific networks have been shown to be more useful for dissecting pathogenesis, since most human diseases are tissue-specific^18^. Using mass spectrometry coupled to liquid chromatography (LC-MS) to analyze tagged proteins and heterologous expression in cultured cells, thousands of novel PPIs have been reported^19,20^. These herculean studies have provided important biological insights, but this data often does not accurately reflect the uniqueness and complexity of brain tissue. In addition, heterologous expression of proteins with ectopic tags has been reported to alter the native characteristics of endogenous proteins, which could affect their PPIs^21,22^. Immunoprecipitation (IP) of endogenous proteins with antibodies (Ab) has also been used to identify PPIs, which is more amenable to analysis in tissue and ultimately animal models of disease^23^. For example, a recent LC-MS study using an Ab to the endogenous Akt kinase discovered a novel functional interaction between Akt and potassium/sodium hyperpolarization-activated cyclic nucleotide-gated channel (HCN1) in the rat hippocampus^24^. This interaction was not identified in two recent large-scale PPI datasets in cultured cells using tagged Akt as a bait, suggesting that it is a brain-specific interaction^19,20^. Quantitation of PPIs between different physiological states is also needed to understand the differences in signal transduction under normal and diseased conditions. PPIs are emerging as attractive targets for drug development^25,26^ yet reports of accurate quantitation of IP-LC-MS datasets between disease states in tissue are scarce. Regardless of whether Ab or tagged proteins are used to identify PPIs from a biological sample, transient PPIs are poorly recovered, and it cannot be determined whether the identified interactor binds directly to the target (i.e., bait) protein or resides in a complex of proteins that include the target protein (i.e., indirect). Protein complexes are essential to cellular biology to allow efficient signal transduction. Proteins need to navigate through an intricate proteome to form the appropriate PPIs with precise temporal and spatial accuracy. Protein evolution has alleviated this problem through the formation of signaling complexes, which are organized by adaptor or scaffolding proteins^27–29^. For example, kinases and phosphatases with different substrates can exist in a protein complex for precise regulation of phosphorylation; however, these kinases and phosphatases do not form direct PPIs. In the brain, the postsynaptic density, which regulates synaptic plasticity, contains 1500 diverse functional proteins (i.e., receptors, ion channels, kinases, scaffolds, phosphatases, and cytoskeletal proteins) constituting a collection of protein complexes^30^. Thus, indirect PPIs are indicative of proteins residing within multiprotein complexes, which are required for localized signal transduction throughout the proteome. Manipulation of protein complexes via scaffolding proteins has therapeutic potential^31^.

This study used an IP-LC-MS strategy to construct a brain-specific SCZ PPI network by identifying the PPI of multiple proteins implicated in SCZ. Eight SCZ risk factors were immunoprecipitated from the rat hippocampus, a brain region vulnerable to SCZ pathology^32^. The hypofunction of glutamate has been hypothesized to trigger the pathogenesis of SCZ^33^. This hypothesis is supported by the finding that the synaptic network enriched from SCZ risk genes is intimately connected to N-methyl-D-aspartate glutamate receptors (NMDAR) function^34–36^. Since many SCZ symptoms are uniquely human, there is no single animal model that truly replicates all the complex human SCZ phenotypes^37^. In this respect, all SCZ animal models can be considered limited. Phencyclidine (PCP), a non-competitive inhibitor of the NMDAR, induces SCZ symptoms in humans and exacerbates symptoms in SCZ patients^38^. Animals treated with PCP have been used to dissect the neurobiology of SCZ and to successfully screen for SCZ drugs^39,40^. NMDAR complexes are highly conserved between rodents and humans^41,42^, which accounts for the remarkably similar responses to PCP. To gain insight into SCZ pathogenesis, we quantified the effect of PCP on our PPI network. PCP was administered for <30 min, which precluded any changes in transcription or translation, thus allowing us to focus on PPI.

## Experimental procedures

### Animals

Sprague–Dawley male rats (Harlan Laboratories, San Diego, CA, USA) weighing 300–400 g were injected with either saline or PCP (1.25 mg kg^–1^; Sigma Aldrich, St. Louis, MO, USA) at a volume of 1 ml kg^–1^. After 26 min, rats were euthanized and brains dissected as previously described^43^. Sprague–Dawley rats were labeled with ^15^N as previously described^43^. Animals were housed in pairs in clear plastic cages located inside a temperature- and humidity-controlled animal colony and were maintained on a reversed day/night cycle (lights on from 7:00 P.M. to 7:00 A.M.). Food (Harlan Teklad, Madison, WI) and water and food ad libitum. Animal facilities were AAALAC-approved, and protocols were in accordance with the “Guiding Principles in the Care and Use of Animals” (provided by the American Physiological Society) and the guidelines of the National Institutes of Health.

### Immunoprecipitations

Each hippocampus was homogenized on ice in 4 mM HEPES, pH7, 200 mM NaCl with protease and phosphatase inhibitors with an Eppendorf dounce grinder. The IP were performed as previously described^24^. Briefly, IP buffer(4 mM HEPES, pH7, 200 mM NaCl, 0.5% Triton, 0.5% NP-40, and 0.01% Sodium Deoyxcholate) was added to the homogenates and incubated overnight. The homogenates were centrifuged for 30 min at 17,000 × *g*. Normal serum bound to Protein A(Life Technologies) or Protein G (Abcam) beads was added and incubated overnight. The precleared supernatant was divided into two Eppendorf tubes, and either beads crosslinked to the bait antibodies or normal sera. Antibodies employed: Grin2b-rabbit #06-600 Millipore,Ppp1ca- Abcam Rabbit #ab16446, Syngap - rabbit #ab3344, Stx1a – Abcam rabbit #ab170890, Map2k1 – Abcam rabbit #ab32091, Syt1 – Abcam mouse #ab13259, Grm5 – Abcam mouse #ab134271, Gsk3b – Abcam mouse #ab2602. Controls used the normal sera of the same species as the target antibody. Mouse antibodies were crosslinked to Protein G beads, and rabbit antibodies were crosslinked to Protein A beads. The beads were incubated with supernatants overnight and then washed three times with 4 mM HEPES, pH 7, 200 mM NaCl. The beads were frozen at −80 °C until protein digestion. All procedures were performed on ice or at 4 °C. For immunoblot analysis of IPs, the same protocol was followed except the beads were eluted for 10 minutes at 70 °C with 4X Laemmli buffer.

### Cell culture

Cells were grown in a 37 °C incubator with 5% CO2. HEK cell media consisted of DMEM containing 10%FBS with 100 IU/ml penicillin and 100 *μ*g/ml streptomycin, and N2A cell media consisted of EMEM containing 10%FBS with 100 IU/ml penicillin and 100 *μ*g/ml streptomycin. Cells were transfected with XtremeGene 9 according to the manufacturer’s protocol. cDNAs transfected were either a rat Grm5 with a C-terminal Myc tag (Origene#RR201568) C-terminal, rat Grm3 with a C-terminal Myc tag (Origene# RR208773), or untagged mouse BK-alpha, which was a gift from Robert Brenner (Addgene plasmid # 113566)^44^. Cells were harvested 2 days after transfection and lysed in the 4 mM HEPES, pH 7.4, 200 mM NaCl, 0.5% NP-40, and 0.5% Triton. Lysates were centrifuged 17,000 × *g* for 10 min, and then a BCA assay was performed on the supernatant. Supernants were immunoprecipitated with Myc agarose beads (Bethyl Laboratories, S190-104). Beads were washed with the lysis buffer without detergent, and proteins were eluted for 10 minutes at 70 °C with 4X Laemmli buffer.

### Immunoblot analysis

Samples were separated with a 3–8% Tris-Acetate gradient gel(Life Technologies), transferred to PVDF blotting paper, and developed using Supersignal Chemiluminescence Substrate (Thermo Scientific) with the Azure c600 Biosystems developer as previously described. Immunoblots were incubated with the following primary antibodies: anti-Kcna4 (Antibodies, Inc 75-010), anti-Stx1a (SantaCruz Antibodies, sc-12736), anti-Kcnma1 (Antibodies, Inc *75-408)* and anti- Myc (Cell Signalling, 71D10). Immunoblots were then incubated with secondary antibodies conjugated to HRP (JacksonImmuno)^24^.

### Protein digestion

Proteins were eluted from the beads twice with 5% SDS, then the eluates were heated at 90 °C for 10 min. The eluate was precipitated with methanol and chloroform. The precipitate was digested with trypsin and ProteaseMAX (Promega) as previously described^43^.

### Liquid chromatography coupled to mass spectrometry (LC-MS) analysis

Each digestion was analyzed twice by mass spectrometry to create two technical replicates per IP. For each technical replicate, 15 μL of the digestion mixture was injected directly onto a 50 cm 100 μm i.d. capillary containing 1.7 μm BEH C18 resin (Waters). Peptides were separated at a flow rate of 300 nl/min on Easy-nLC 1000 (ThermoFisher) on a 220 min reversed-phase gradient. Peptides were eluted from the tip of the column and nano electrosprayed directly into an Elite or Velos Pro mass spectrometer (ThermoFisher) by application of 2.5 kV voltage at the back of the column. The Grin2b IP was analyzed by MudPIT as previously described^45^. The mass spectrometer was operated in a data-dependent mode. Full MS1 scans were collected in the orbitrap at 240 K resolution, and 20 rapid CID MS2 scans in the ion trap. Dynamic exclusion was used with an exclusion duration of 30 seconds.

### Interpretation and quantitation of LC-MS data

Data from technical replicates were combined prior to database searching. MS2 (tandem mass spectra) were extracted from the XCalibur data system format (.RAW) into MS2 format using RawConverter^46^. The MS2 files were interpreted by ProLuCID, and results were filtered, sorted, and displayed using the DTASelect 2 program that employed a reversed-sequence decoy database strategy and 5 ppm mass accuracy filtering^47^. Searches were performed against the UniProt-reviewed rat database with isoforms 03-25-2014. For each LC-MS dataset, the protein FDR was <1%. The ^14^N/^15^N quantitation and statistical analysis were performed by Census, as previously described^43^. Before statistical analysis, the ^14^N/^15^N protein ratios were normalized by the ^14^N/^15^N protein ratio of each target.

### Bioinformatic analysis

Protein interactors for each bait were identified with SAINTexpress using default values; each SAINT dataset was generated from three bait IP and three control IP^48^. The input for SAINT was only the ^14^N identifications. For the comparison with the BioGRID database, the physical interactions for each target were downloaded for mouse, rat, and human experiments. For the comparison of the String database, the “physical subnetwork” option was chosen using the mouse species and the high confidence setting. Enrichment of GO terms was performed by WebGestalt with <0.0001 FDR^49^. To determine if the interactors identified by SAINT have been reported as SCZ susceptibility genes, multiple databases that combine datasets from published reports were used. SCZ GWAS database (ID# MONDO_0005090) was downloaded from the NHGRI-EBI Catalogue of human genome-wide association studies. De novo mutation datasets and datasets of genes differentially methylated in SCZ were downloaded from the SZDB2.0^50^. All data was downloaded in December 2025. The heatmap was created with www.heatmapper.ca using the average linkage clustering method and the Euclidean distance. Figure 4d was created with CIG-P^51^, and other figures were created with Photoshop, Microsoft Excel, GraphPad Prism, and Biorender.

**Fig. 1:**
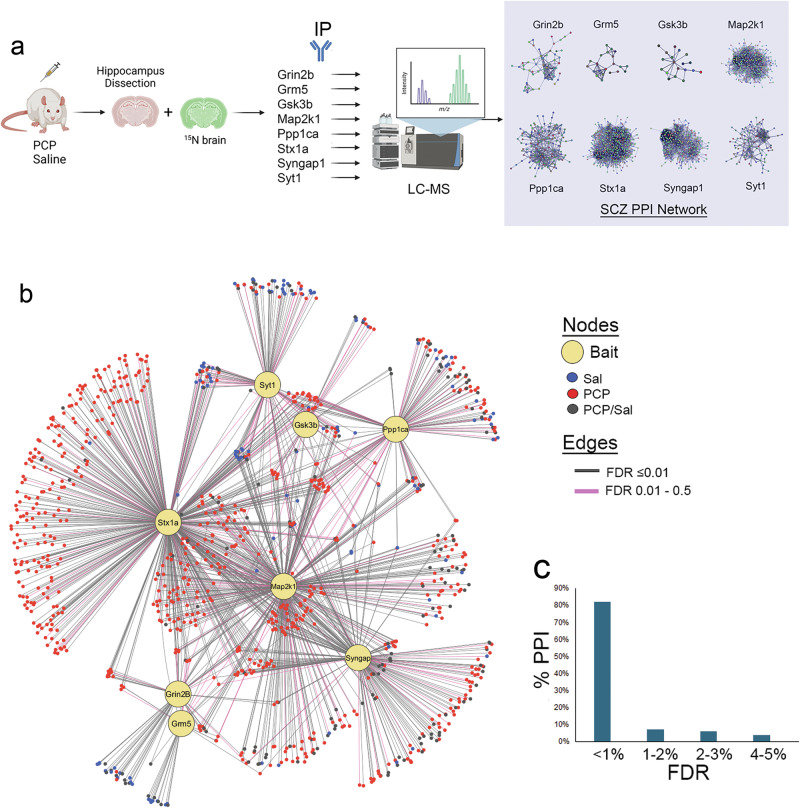
Construction of a SCZ PPI network. **a** Workflow used to generate the SCZ PPI network. **b** Interaction network of the 1612 PPI identified in this study. The eight large yellow nodes represent the baits (i.e., target proteins) and the smaller nodes represent the 1612 PPI. The color of each PPI node represents if was identified from saline (blue), PCP (red), or both conditions(black). The FDR of each PPI is represented by black (FDR ≤1%) or pink (>1%) edges. **c** The distribution of the FDR for the 1612 PPI that is depicted in **b**.

## Results

### Selection of SCZ susceptibility genes

Our goal was to identify the PPI networks of SCZ susceptibility genes in vivo. We chose eight SCZ susceptibility genes or targets to construct our SCZ PPI network based on numerous criteria. First, we consulted comprehensive SCZ susceptibility gene databases (i.e. SZDB2.0 and SZGR2^50,52^) that have compiled published studies analyzing large SCZ cohorts employing a variety of genetic methods, including GWAS, de novo mutation analysis, and differential methylation analysis, which have implicated thousands of genes in SCZ. Second, we searched for targets with a synaptic localization since synaptic dysfunction is a central hypothesis in SCZ^34^. Third, since phosphorylation is required for many PPI, we looked for phosphorylation changes induced by PCP in our targets^43^. However, the major limiting factor of our study was the identification of commercial antibodies capable of IP-LC-MS. These criteria resulted in 8 targets implicated in SCZ through expression or activity changes in human and animal SCZ samples or genetic association: Grin2b^53–55^, Grm5^56–58^, Gsk3b^59–62^, Map2k1^11,63,64^, Ppp1ca^43,65,66^, Stx1a^67–69^, Syngap1^70,71^, and Syt1^67,72^. A PubMed literature search revealed that several laboratories have published reports on the involvement of these targets in SCZ pathogenesis (Supplementary Fig. 1a). For example, Grin2b is a subunit of the NMDAR, whose dysfunction is central to the glutamate hypofunction SCZ hypothesis. The targets are functionally diverse but are interconnected in many ways beyond their potential involvement in SCZ pathogenesis. (Supplementary Fig. 1b). For example, Grin2b, Grm5, Map2k1, and Gsk3b are listed as PPI of Syngap1 in the BioGRID (The Biological General Repository for Interaction Datasets) database^73^. Functional interactions of the targets include the regulation of Grm5 activation by NMDAR^74^.

### Novelty of the SCZ PPI Network

**Fig. 2:**
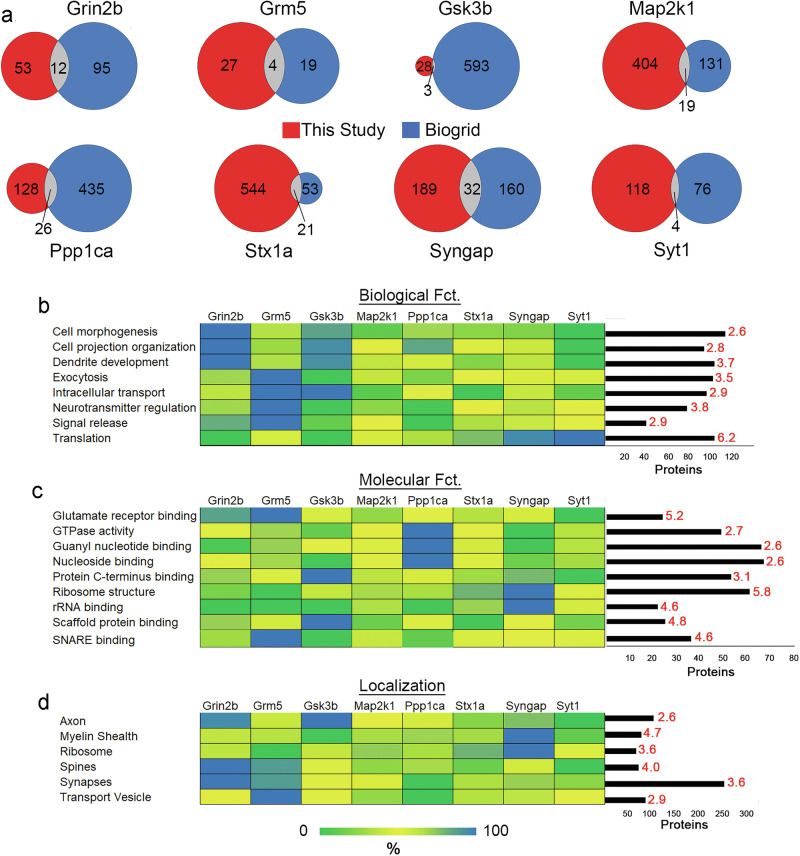
Biological pathways in the SCZ PPI network. **a** Comparison of the interactors of each bait in this study and BioGRID. For this comparison, Sal and PCP PPI were combined into a unique list. Identification of significant GO enrichment terms of the SCZ disease network for three analyses: **b** biological function (*p*<2.22e^−6^), **c** molecular function (*p*< 1.28e^−10^), **d** cellular component or localization (2.2e^−16^). The bar graph depicts the number of interactors assigned to each enriched GO term. The red number above each bar represents the enrichment value over the background whole brain proteome. The heatmap depicts the percentage of interactors from each bait annotated to the enrichment GO term.

Figure 1a depicts the experimental workflow. The rats were injected with 1.25 mg/kg of PCP or saline (SAL) and were sacrificed after 26 min. The hippocampi were dissected. These unlabeled or 14 N hippocampi were homogenized then mixed 1:1(wt/wt) with 15 N labeled rat brain homogenate to serve as an internal standard for the quantitation of PCP and SAL interactomes for LC-MS^75^. All the targets are localized to the synapse but can also be found in non-synaptic compartments and expressed in non-neuronal cells. Thus, since there is also evidence for non-synaptic perturbations contributing to SCZ pathogenesis, we chose to perform an unbiased analysis on unfractionated brain tissue^76^. The endogenous targets were immunoprecipitated (IP) with antibodies from three PCP rats and three SAL rats. In parallel, six control IPs (i.e., 3 PCP and 3 SAL) were performed. In total, 96 (48 bait and 48 control) IPs were performed, and each was analyzed separately by LC-MS analysis. Protein identifications were similar between the PCP and SAL samples but varied between the different targets (Supplementary Fig. 1c,d). The target proteins were enriched at least 9-fold compared to control IPs and there were no significant differences in the target abundance between SAL- and PCP- treated brains (Supplementary Fig. 2a). The proteins identified in the target IPs were analyzed by the computational tool SAINT (Significance Analysis of Interactome), which determines the probability of a protein interacting with the target by comparing its abundance distribution to the control IP^77^. This analysis was performed on the 14 N identifications, and PCP and SAL datasets were analyzed separately. There were 1612 PPI observed using a 5% FDR in SAINT(Fig. 1b, c). Although we are using the term “PPI”, we cannot determine if these proteins are directly interacting with a target or part of the same protein complex (i.e. indirect). Some interactors were classified as a PPI to multiple targets, but the majority (58%) were specific to one (Supplementary Fig. 2b, c and Supplementary Tables 1–3). When all the datasets were combined, we identified 1007 unique proteins as PPI to at least one target protein and 199 (20%) of these PPI were determined to have a transmembrane domain. In terms of translational relevance, there is evidence that 96% of our SCZ network is expressed in the human hippocampus^78–80^. This PPI dataset was compared to physical protein interactions deposited in the BioGRID database, which includes reports employing a wide variety of low- and high-throughput PPI techniques. There were 713 publications in BioGRID that described at least one interaction with one of our targets, and these were compared to our dataset (Supplementary Table 4). All targets had a PPI that was verified by BioGRID, but 92% of our PPI were not observed in BioGRID. (Fig. 2a). We also examined the String database using the physical complex option^81^. String confirmed BioGRID or verified fewer PPI in our network with all the targets except Stx1a. String verified nine additional PPI in the Stx1a PPI dataset. In total, we validated 133 previously reported PPI. We verified two novel PPI by immunoblots. Potassium voltage-gated channel subfamily A member 4 (Kcna4) was validated in Grin2B brain immunoprecipitates (Supplementary Fig. 3a), and syntaxin-1A (Stx1a) was validated in Grm5 immunoprecipitates (Supplementary Fig. 3b). We also validated the novel PPI between the calcium-activated potassium channel subunit alpha-1 or Kcnma1 (i.e. Bk-alpha channel) and GRM5 with heterologous expression in cultured cells (Supplementary Fig. 3c, d). We observed Kcnma1 in the immunoprecipitates of GRM5 tagged with Myc, but absent in the immunoprecipitates of another metabotropic glutamate receptor GRM3 tagged with Myc.

**Fig. 3:**
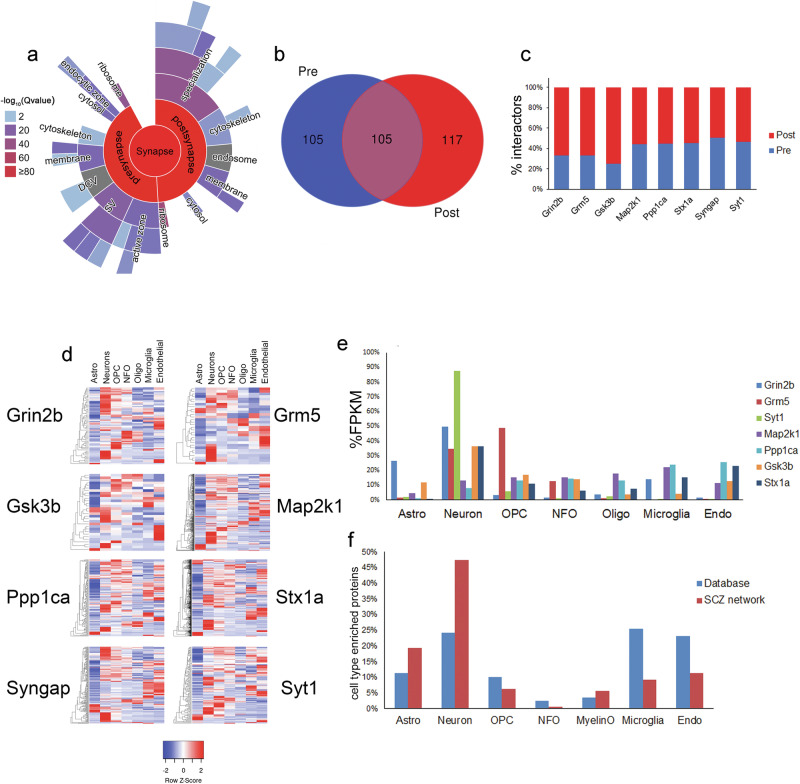
Multiple cell types exist in the SCZ PPI network. **a** Significant enrichment of synaptic subcellular compartments in the PPI network. Colors represent *p-*values of the significant enrichment **b** Overlap of pre- and post-synaptic proteins in **a**. **c** The percentage of proteins exclusively pre- and post-synaptic proteins assigned to different baits. **d** Heatmap of the RNA expression of protein interactors in different brain cell types. Astro Astrocytes, OPC oligodendrocyte precursor cells, NFO newly formed oligodendrocytes, Oligo myelinating oligodendrocytes). **e** RNA abundance distribution of our targets in different brain cell types. **f** Percentage of protein interactors that were enriched in one brain cell type compared to the entire brain RNA database.

### Biological Interpretation of SCZ PPI network

The significant enrichment of GO terms was determined for the entire SCZ PPI network (Fig. 2b–d). The enriched biological functions included dendrite development, neurotransmitter regulation, intracellular transport, and translation. At the molecular level, glutamate receptor binding, SNARE binding, scaffolding binding, and ribosome structure were significantly enriched. The frequency of these functionally annotated interactors varied among the different targets. The Grm5 network had the highest percentage of interactors annotated to intracellular transport. This corresponds to reports that describe Grm5 as an atypical GPCR with more intracellular localization than plasma membrane localization^82,83^. Syngap1 had the highest percentage of interactors involved in translation and ribosome structure regulation, which correlates with its regulation of protein synthesis^84,85^. The enrichment of interactors annotated to scaffolding binding suggests the identification of protein complexes. Many different cellular components were also significantly enriched. Transport vesicles and ribosomes were enriched, consistent with the enriched biological functions. The cellular compartment with the most annotated proteins was the synapse. Synaptic interactors were observed in all the interactomes, but Grin2b interactome had the highest percentage. These synaptic proteins were validated and further investigated with the SynGo synaptic database^86^. This database mapped 41% (418 proteins) of the PPI to unique synaptic genes, which was calculated as a significant enrichment (*p* value = 2.6 e-175) (Fig. 3a and Supplementary Table 5). In addition, 35 sub-synaptic child terms were significantly enriched with a 1% FDR. These annotated synaptic interactors were evenly distributed between the two largest sub-synaptic compartments, the pre- and post-synapse (Fig. 3b). The SynGo database assigns a postsynaptic localization to all the targets except for Syt1, which was not designated to either compartment. Grin2b, Gsk3b, Ppp1ca, and Stx1a were also annotated to the pre-synapse. The PPI annotated exclusively to the pre- or post-synapse were evenly distributed among the targets except Grin2b, Grm5, and Gsk3b, which had more assigned to the post-synapse (Fig. 3c). Thus, this data demonstrates that many of these novel PPI may occur at the synapse. Non-synaptic interactors represented 59% of our network, suggesting that some PPI may occur in non-neuronal cells. Although RNA and protein levels do not always correlate, we annotated our network with a transcriptome rodent brain database of eight cell types^87^, which has been employed previously to extract cell type information from proteomic datasets^88,89^. All the targets were detected in all cell types except Syngap1 which was not present in the transcriptome database (Fig. 3e). We also observed a large variation of cellular distributions for the interactors (Fig. 3d). Next, we sought to determine how many interactors are enriched in a particular cell type by conservatively defining cell enrichment as a protein with >50% RNA signal in one cell type. We identified 175 PPIs enriched in one cell type, whereas the entire database had 4008 proteins enriched (Fig. 3f). We observed that 47% of the enriched protein interactors were in neurons, whereas only 24% of the enriched proteins in the entire database were in neurons, which is consistent with the observed enrichment of synaptic interactors. We also observed an increase in protein interactors enriched in astrocytes compared to the database. Overall, this analysis provides evidence that our identified PPI may occur in non-neuronal cells in addition to synapses.

**Fig. 4:**
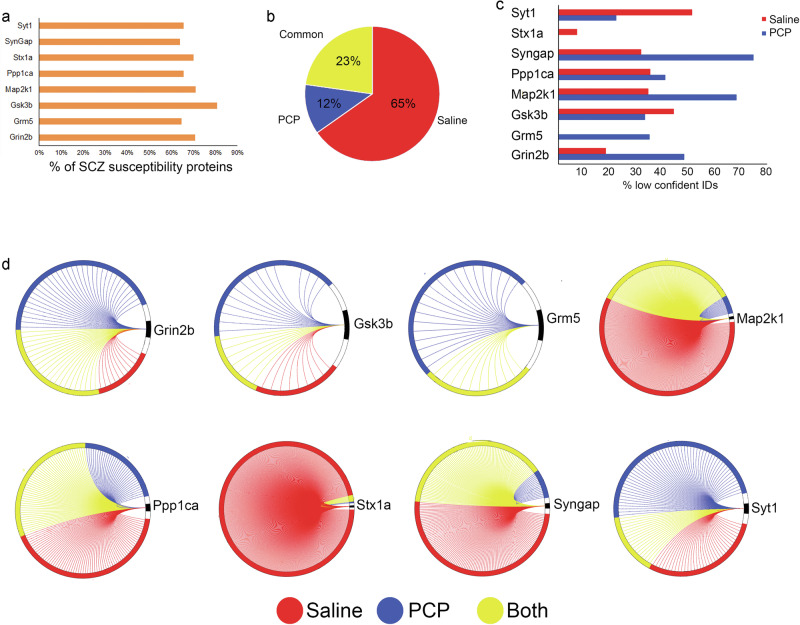
The effect of PCP on the SCZ PPI network. **a** Percentage of PPI assigned to each target that has evidence of being a SCZ susceptibility protein. **b** The pie graph represents the percentage of PPI identified by SAINT in PCP, saline, or both drug datasets for all the baits. **c** Percentage of PPI with a 20% SAINT FDR in one drug condition and a 5% FDR in the other drug condition. **d** Each circle represents one bait (black) and each line represents a unique interactor for that bait. The color of each line represents interactors that were identified in PCP (blue), Saline (red) or both datasets (yellow) by SAINT.

It has been reported that SCZ susceptibility proteins interact with one another beyond the level expected by chance^15^. The targets have been reported as SCZ susceptibility genes by several published studies analyzing large SCZ cohorts employing a variety of genetic methods, including GWAS, de novo mutation analysis, and differential methylation analysis. Cross-referencing these published genetic studies with our PPI network, 69% (698) of our PPIs have previously reported evidence of being a potential SCZ risk factor (Fig. 4a). Thus, our SCZ network corroborates the theory that SCZ susceptibility proteins form a physical interaction network.

### PCP modulates the SCZ PPI network

We determined how PCP affected the identification of a highly confident PPI. First, the distribution of PPI between PCP and SAL networks was compared. Sixty-five percent of the PPI were only observed in SAL, and only 12% were specific to PCP brains (Fig. 4b). This suggests that PCP causes a widespread disruption of native PPI. However, this trend was not observed with all the targets (Fig. 4d). The majority of PPI for Grin2b, Grm5, Gsk3b, and Syt1 were observed in PCP and not SAL, suggesting that PCP induces novel PPI for these proteins. Next, the SAINT datasets were re-examined with less stringent filtering (i.e., 20% FDR). We found that 36% of the high confidence PPI specific to one condition at 5% FDR were also observed in the other condition when using the lower confidence threshold of 20% FDR (Fig. 4c). This suggests that PCP may weaken or enhance some PPI rather than completely turning them “on” or “off”.

**Fig. 5:**
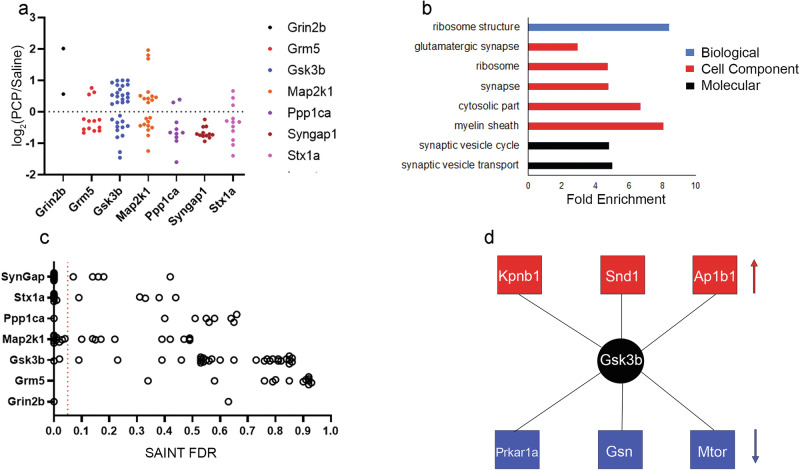
SILAM analysis of the SCZ PPI network. **a** Altered proteins (*p* < 0.05) between PCP and SAL LC-MS-IP datasets. **b** Significantly enriched (*p* < 0.0002) GO terms from the protein altered in (**a**). **c** Altered proteins in 5a correlated with their SAINT FDR. The red dotted line indicates SAINT FDR 0.05. **d** Gsk3b interactors in the BioGRID database and significantly altered in the Gsk3b SILAM experiment in 5a, but not assigned as a Gsk3b PPI in the SAINT analysis. Red indicates upregulated, and blue indicates downregulated with PCP.

To further investigate the effect of PCP, the direct differences between SAL and PCP datasets for each target were quantitated by employing the 15 N internal standard. Significant changes induced by PCP were observed for all targets except Syt1 (Fig. 5a and Supplementary Table 6). Overall, the network PPI decreased by 60% with PCP treatment, but Grin2b, Gsk3b, and Map2k1 did not follow this trend, instead demonstrating that most PPIs increased with PCP. The enriched GO terms were mirrored those in the SAINT analysis but included GO terms involved in synaptic biology (Fig. 5b). Twenty-seven percent of the proteins significantly altered by PCP were also deemed a PPI by SAINT (FDR < 0.05) (Fig. 5c). Interestingly, six significantly altered proteins are reported as interactors with Gsk3b by the BioGRID database but not identified as a PPI with our SAINT analysis (Fig. 5d). This suggests adding a quantitative dimension to PPI mapping aids in identifying interactors.

### Prediction of direct interactions with target proteins

**Fig. 6:**
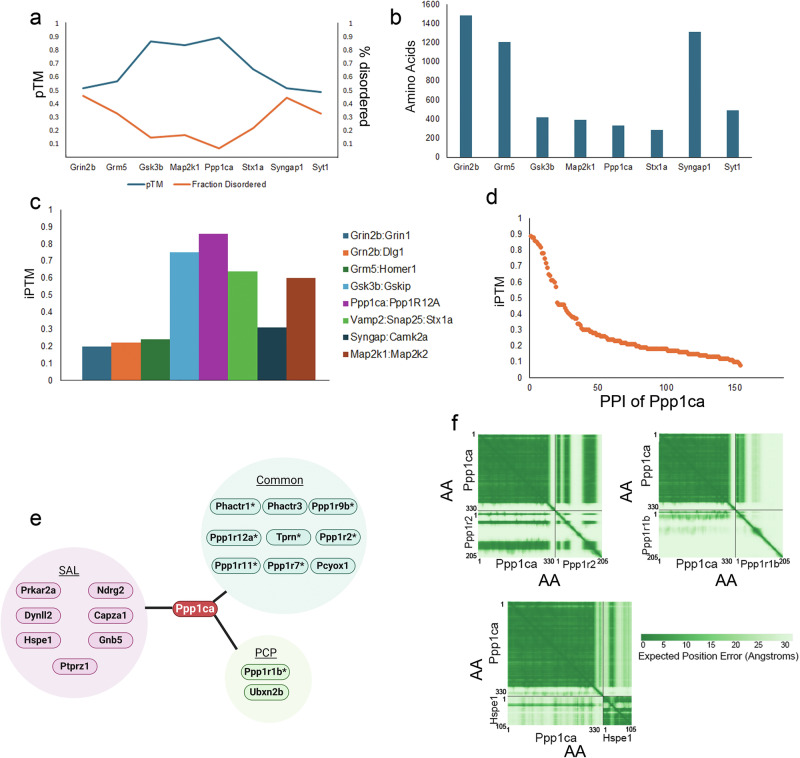
AF3 identifies potential direct PPI. **a** pTM and fraction disordered scores assigned to target proteins by AF3. The left y axis indicates pTM values, and the right *y* axis represents the percentage fraction disordered. **b** The number of AA of each target protein. **c** iPTM scores assigned to known PPI of the target proteins. **d** iPTM score for each of the 154 PPIs assigned to Ppp1ca in this study. **e** PPI from D that had an iPTM ≥ 0.6. * indicates the interactor has previously been reported to bind directly to Ppp1ca. **f** AF3 PAE plots aligning the AA of Ppp1ca with Ppp1r2, Ppp1r1b, and Hspe1. Green represents the confidence of the relative orientation between two AA, with the dark hue representing more confidence. Each protein AA is plotted from the N- to the C-terminus, with the first and last AA listed numerically on each plot.

A disadvantage of LC-MS studies with tag or antibody protein enrichment is that it cannot distinguish between a PPI that binds directly to the target protein, and a PPI in which the interactor and target protein reside in the same multiprotein complex (i.e. indirect). We sought to predict which PPI may be directly interacting with their target proteins by using the artificial intelligence algorithm AlphaFold3(AF3)^90^. First, we analyzed the predicted AF3 structure of the targets using the pTM score and determined the fraction of each structure that was calculated to be disordered (Fig. 6a). Our reasoning was that if our targets have poorly resolved structures, then it will be difficult to screen for direct PPI. A pTM score >0.5 suggests that the structure may be correct, with the highest confidence equaling 1. Undefined or disordered regions hinder the accuracy of the prediction. All our targets possessed a pTM score > 0.5 except Syt1. The fraction of disordered negatively correlated with the pTM score, as expected. Gsk3b, Ppp1ca, and Map2k1 were the target proteins with the highest pTM scores and were also the smallest of our targets (Fig. 6b). Ppp1ca had the most confident structure (i.e. pTM 0.9) and the least fraction disordered (i.e. 0.07). Next, we determined the AF3 prediction of previously reported direct interactions of the targets that were also observed in the PPI network. We used the iPTM score to determine the confidence of the interaction. An iPTM score >0.8 is a highly confident direct interaction, whereas <0.6 is a failed prediction, which is evidence against a direct interaction. In general, AF3 failed to identify a highly confident direct interaction of targets with low pTM scores and larger disordered fractions (Fig. 6c). Ppp1ca is the only target where its known direct interactor was deemed a highly confident interaction. Thus, we chose to analyze our Ppp1ca PPI dataset with AF3. We screened the 154 PPIs that were assigned to Ppp1ca (Fig. 6d and Supplementary Table 7). Eight interactors had an iPTM score >0.8. These eight PPIs have previously been reported to form a direct interaction with Ppp1ca, except Phactr3. Phactr3 is structurally similar to, but less studied than, the reported direct interactor, Phactr1. These interactors are all inhibitors of PP1 except for Ppp1r9b, which targets Ppp1ca to specific subcellular compartments^91^. Ten PPIs were assigned a score <0.8 and ≥0.6. This included the known direct interactor Ppp1r1b (aka DARPP-32)^92^. Ubxn2b (with an iPTM score of 0.6) has been reported to be an interactor of Ppp1ca, but it was not investigated whether it was a direct interaction^93^. Among the 18 interactors predicted to bind directly to Ppp1ca, 50% were identified with both SAL and PCP, whereas 2 (i.e. 11%) were only identified with PCP (Fig. 6e). Ppp1r1b was one of the direct interactors that was only observed with PCP, and it was previously reported that PCP activates its phosphatase inhibitory activity (16). The PPIs with iPTM scores ≥ 0.6 were analyzed with another complex prediction algorithm, Boltz-2 (Supplementary Fig. 4a)^94,95^. Most of the iPTM scores were less than AF3, including the previously reported direct PPI. The Ppp1ca possesses over 200 PPIs, but its docking motifs are too small and degenerate to allow for successful screens of our candidates^96^. Its multiple docking sites allow for a single interactor to bind to multiple sites or multiple proteins to bind simultaneously^97,98^. The known direct Ppp1ca interactors in our data possessed distinct patterns in their PAE (Predicted Aligned Error) plots, which correspond to structural studies (Fig. 6f and Supplementary Fig. 4b–f). For example, Ppp1r2 interacts with Ppp1ca at three sites (i.e. 12-17AA, 44-56AA, and 130-169AA), but it is only the N-terminus (7–35 AA) of Ppp1r1b that interacts with Ppp1ca^99,100^. Of the novel PPI with iPTM scores ≥ 0.6, we only observed a discrete confident binding region in the PAE plot of Hspe1 (Fig. 6f and Supplementary Fig. 4g–j). Hspe1 is a chaperone involved in protein complex assembly and regulates cell signaling^101^. Hspe1 and Ppp1ca have overlapping localizations with both residing in the cytosol and mitochondria^102^. It has been demonstrated that chaperones are needed for the correct assembly of the PP1 protein phosphatase complex^103^. Our data suggests that Hspe1 may provide another mechanism for PP1 phosphatase assembly.

## Discussion

We performed in vivo PPI network mapping of eight targets that have been implicated as SCZ risk factors. We validated 133 previously reported PPIs, which represent less than 10% of our PPI network. The large number of novel PPI likely stems from the scarcity of IP-LC-MS experiments previously performed in brain tissue, and none have previously been performed in a SCZ animal model. At least 713 publications in BioGRID reported a PPI with at least one of our targets, and 148 were high-throughput unbiased experiments. Only 9% (13) of these high-throughput experiments were performed in brain tissue and only one of these experiments shared a target (i.e., Syngap1) with our study(12). Correspondingly, our Syngap1 dataset did have the largest overlap with BioGRID. However, our network has important methodological considerations. Our network is biased by the antibody chosen for target enrichment. Depending on the specific antibody-target interaction, the capture of the bait could be affected by PTMs or the structure of the target. Thus, some of our PPI may be specific to target proteoforms recognised by our antibody. Furthermore, our SCZ network contains both direct binary physical interactions and “co-complex” or indirect interactions. For example, our SCZ network validated the interaction between Grm5 and Homer, which directly binds the C-terminal tail of Grm5^104^. IP-MS analysis cannot distinguish between these two interaction types unless previously reported or until further experiments are performed. To address this challenge and provide evidence for direct interactors, we employed the AF3 algorithm to screen the PPI of Ppp1ca. Our AF3 filter settings may be too conservative and generate false negatives; another study employed a more lenient 0.3 filter followed by additional experimental interrogation to screen for direct PPI^105^, suggesting that interactions falling between our threshold and 0.3 could represent valid direct interactions requiring further validation. Upon further investigation of the AF3 data, Hspe1 was the most confident novel direct interactor of Ppp1ca, which we propose regulates the structure and PPI of Ppp1ca that has been observed by other chaperones. With over 100 validated PPIs, we demonstrate the accuracy of our pipeline. Our data indicates that IP-LC-MS studies in brain tissue have a huge potential to uncover novel PPI for a greater molecular understanding of neurobiology and to improve in silico network analyses for neurological diseases. Even though our network was constructed in the context of SCZ, our dataset has relevance for other neurological diseases where our targets have been implicated.

There were similarities between our in vivo SCZ PPI network and in silico SCZ genetic networks. First, we observed the enrichment of synaptic proteins. Using the SynGo database, 418 interactors (41% of our network) were identified as synaptic, consistent with synaptic localization of these targets. Defining the synaptic proteome is inherently difficult because the synapse is an “open organelle”, and many synaptic proteins also have non-synaptic localizations and are expressed in non-neuronal cells. We further attempted to define our synaptic PPI by differentiating between pre- and postsynaptic compartments via SynGo. Half of our targets were annotated to both compartments, and all targets had PPIs that were annotated to both. IP-MS analysis of SynGAP from the mouse hippocampal postsynaptic density has been reported^106^ and we confirmed 27 of their reported PPIs, further confirming a postsynaptic localization of some of our PPIs. This data supports the emerging evidence against the canonical localization exclusivity of the pre and post synapse^107,108^. However, the exact aetiology of SCZ remains unclear and synaptic dysfunction is only one hypothesis^109^. There is evidence for the involvement of non-neuronal cell types, including endothelial cells, astrocytes, and microglia^110,111^. Since more than half of our PPI currently have no known synaptic localization, our study supports the role of non-synaptic mechanisms underlying SCZ pathogenesis. Comparing our data to RNA, our network was enriched with proteins localized to neurons and astrocytes. SCZ patients have an increased activation of astrocytes, and astrocytic genes are significantly associated with SCZ patients^76^. PCP causes the activation of astrocytes and increases the expression of astrocytic glutamate transporters, which induces increased glutamate uptake and reduces extracellular glutamate^112^. Astrocytes are involved in multiple brain functions, including immune response, homeostasis, nutrient permeability, and synaptic remodeling. It is unclear how astrocyte dysfunction contributes to SCZ pathogenesis. One hypothesis is that the release of D-serine from astrocytes is compromised^113^. D-serine binds NMDAR and is required for activation, which supports the hypofunctional glutamate SCZ theory. Alternatively, since astrocytes can regulate synapses^114,115^, synaptic dysfunction and perturbations in non-neuronal cells in SCZ etiology are not mutually exclusive. Our data support emerging evidence that pathogenesis is multifaceted and involves dysfunction in multiple cell types.

Our PPI network demonstrates high confidence through multiple lines of validation, including over one hundred PPI validated, as well as numerous novel interactions with potential relevance to SCZ pathophysiology. The observation of numerous reciprocal PPI verified by two different targets further confirms the credence of our SCZ network. For instance, Gsk3b and phosphatase PP1 have been reported to interact^116^. Gsk3b was identified as a Ppp1ca interactor, and two PP1 subunits (Ppp1cc and Ppp1r2) were identified as interactors of Gsk3b. Similarly, members of the synaptotagmin protein family were identified as interactors of Stx1a (i.e., syntaxin-1A) and members of the syntaxin protein family were identified as interactors of Syt1, thus confirming their localisation to a multi-protein complex essential for neurotransmission and vesicular fusion^117^. The Syngap1-Map2k1 interaction that we describe here was originally reported with another LC-MS-IP experiment, with Syngap1 as the target but using a different Syngap1 antibody^106^. But for the first time, we also identified this PPI with a Map2k1 antibody. The function of this reproducible PPI is unknown.

This study also discovered a novel interaction between two targets: Grm5 and Stx1a. This PPI was identified in both the Grm5 and Stx1a datasets and validated by an immunoblot. Stx1a has been co-localized to Grin2b and Dlg4 at the hippocampus postsynaptic density, and it has been posited that Stx1a regulates the trafficking of Grin2b^118^. Grm5 is structurally linked to Grin2b via the scaffolding proteins Homer and Dlg4. Activation of Grm5 can potentiate NMDAR activity, and NMDAR activation can desensitize Grm5^119,120^. This functional relationship has been proposed as a therapeutic target for SCZ^121^. There were 11 interactors identified shared between the Grm5 and Stx1a PPI datasets that are potentially involved in a Grm5-Stx1a complex. Interestingly, one of the interactors is Homer1, and another is syntaxin family member Stx13, which has been reported as a Homer1 interactor^122^. This suggests that Stx1a-Grm5 may be involved in the functional relationship between NMDAR and Grm5.

We also validated two other novel PPI with potassium channels: Grin2b and Kcna4; Grm5 and Kcnma1. In our Grin2b network, we observed three different voltage-gated potassium channel subunits (Kcna4, Kcna6, and Kcnab2), which can form one heterotetrameric channel. Kcna4 can regulate neuronal activity via manipulation of action potentials. Like Grin2b, Kcna4 is localized to the post-synapse and binds to Dlg4^123^. In human studies, changes in the DNA methylation and rare variants of Kcna4 are associated with SCZ patients^124,125^. In addition, Kcna4 has been reported to be upregulated in brain of people who abuse PCP^126^. In mouse studies, it has been reported to be responsible for motivational deficits, which is a symptom of SCZ^127^. It has also been reported to be modulated by the anti-psychotic drug, aripiprazole^128^. Further investigation is needed to determine if the NMDAR hypofunction can alter Kcna4 channel activity, leading to motivational dysfunction. We also validated the interaction between Grm5 and the calcium-activated potassium channel subunit Kcnma1. Functionally, Kcnma1 and Grm5 are intimately involved in the same molecular pathways, providing evidence for a functional interaction. Both proteins regulate pre-pulse inhibition, which is dysfunctional in SCZ patients^129,130^, and both are in a complex with the NMDAR at the postsynapse^131–133^. Kcnma1 variants have been significantly associated with SCZ via GWAS^134–136^ and expression changes have been identified post-mortem SCZ brain tissue^135,137^. Negative modulators of GRM5 intensify the effect of PCP, while positive modulators produce an anti-psychotic effect in animals^138–140^. Positive modulators of Grm5 have been proposed as a drug treatment for SCZ. In this study, we observed a significant decrease in the Grm5:Kcnma1 interaction with PCP. It has been reported that the activation of Kcnma1 is responsible for some effects of Grm5 activation in the spinal cord^141^. Although the anti-psychotic effect of Grm5 is postulated to be through its regulation of the NMDAR, our findings suggest that the SCZ drug potential of Grm5 could involve the regulation of Kcnma1 channels.

We quantitated our SCZ network in response to PCP. Since the PCP treatment is less than 30 minutes, any alteration in transcription or translation can be ruled out, as those processes typically take an hour to be triggered or detected^142^. This suggests that the behavioral changes induced by PCP are mediated by alterations in post-translational modifications, protein trafficking, or PPIs. Indeed, using the protocol employed here, PCP has been reported to alter the phosphoproteome^43^. It is difficult to integrate our PCP network with the PCP phosphoproteome study, since phosphorylation can regulate protein activity, structure, and localization in addition to PPI. Nevertheless, the MAPK signaling pathway was decreased with PCP in the phosphoproteome study, and we observed a decrease in Map2K1 PPI with PCP, suggesting that phosphorylation may regulate the PPI observed in the MAP2K1 network. In this study, we analyzed the effect of PCP in two ways. First, we used the SAINT algorithm to identify PPI for each target with or without PCP. This analysis suggests that PCP weakened many PPIs. For example, Grin2b binds Grin2a to form the NMDAR. Grin2a was observed as a PPI of Grin2b with PCP but not with SAL using a 5% FDR. It was identified as a Grin2b interactor with SAL at 20% FDR. To complement and validate these findings, we employed a second approach. Quantitation using 15 N labeled brains (i.e., Stable Isotope Labeling in Mammals (SILAM)) was also used to study the effects of PCP. In the SAINT analysis, the PPI probabilities were compared between PCP and SAL datasets. In contrast, SILAM was used to directly quantitate proteins in the IPs without determining if a PPI exists, but the data still validated the SAINT results. For example, Dlg1 was observed as a Grin2b PPI with PCP, but not SAL. In the SILAM analysis, there was a significant increase in Dlg1 in the Grin2b IP upon treatment with PCP. Dlg1 binds directly to Grin2a and regulates its trafficking to the synapse^143^. NMDAR agonists increase the phosphorylation of Dlg1, which disrupts this interaction. This reduces Grin2A insertion in the synaptic membrane. Consistent with this report, PCP has been demonstrated to reduce the phosphorylation of Dlg1^43^. Our data suggest that PCP decreases the Dlg1 phosphorylation and drives more Grin2A-Dlg1 to the postsynaptic membrane to form a complex with Grin2b. This hypothesis is also supported by our Grin2b network, where Grin2a was identified as a highly confident interactor (5% FDR) with PCP, whereas with SAL, it was identified as a PPI but with a less robust signal (20% FDR). The improper trafficking of NMDAR has been implicated in cognitive disorders^144^. The effects of PCP on our SCZ network suggest that disruption of NMDAR trafficking may also contribute to SCZ pathogenesis. Supporting this hypothesis, a recent study demonstrated that NMDAR trafficking is altered in three different SCZ models^145^.

Currently, almost 40% of approved drugs target individual receptors, which include GPCRs and ligand-gated ion channels. Many receptor complexes are tissue or cell-specific compared to the individual receptor expression, which allows differential receptor signaling in specific cell types. Thus, targeting complexes could lead to more selective drugs with fewer side effects^146^. Numerous PPI drugs have already been tested in clinical trials^147^. The development of drugs targeting PPI is in its infancy, but it holds great promise for the treatment of neurological disorders because brain regions possess discrete functions and proteomes. The first step in the creation of this exciting drug pipeline is mapping PPI by proteomic studies as described here.

All LC-MS raw files have been deposited at ProteomExchange PXD047042

## Supplementary information

Supplementary Figures and Legends

Supplementary Table 1

Supplementary Table 2

Supplementary Table 3

Supplementary Table 4

Supplementary Table 5

Supplementary Table 6

Supplementary Table 7
