# Supplementary material for: In vivo mapping of protein-protein interactions of schizophrenia risk factors generates an interconnected disease network
Source: Schizophrenia (Heidelb). 2026 Mar 7;12(1):39. doi: 10.1038/s41537-026-00734-1 (PMC13096421; doi:10.1038/s41537-026-00734-1)
Supplement: Supplementary file 1 — Supplementary Figures and Legends [file 41537_2026_734_MOESM1_ESM.pdf]

Supplementary Table Legends

TableS1 –A list of interactors for each bait identified with saline or PCP using a 5% FDR calculated by SAINT. PPI verified by BioGRID or String databases indicated. Each target has its own worksheet within this excel file.

TableS2 – A table of all the interactors (listed as gene symbol) identified in this study and which target they were identified with. Saline and PCP interactors for each bait were combined into one unique list. Interactors highlighted in yellow indicated potential SCZ risk gene, which is plotted in Figure 4A.

TableS3 – One table including all the targets with their PPI and marked to indicate if the PPI was identified in Sal or PCP brains.

TableS4 – List of all the publications from the BioGRID database that was downloaded for each bait protein and were compared to our PPI network.

TableS5- Output of the SynGo analysis. Synaptic Gene Ontology terms assigned to the identified interactors.

TableS6– SILAM analysis of each bait N14/N15 dataset. Significant ($p < 0.05$) proteins are listed by their Uniprot accession number.

TableS7 – All the PPI identified for Ppp1ca and their iPTM score from AF3.

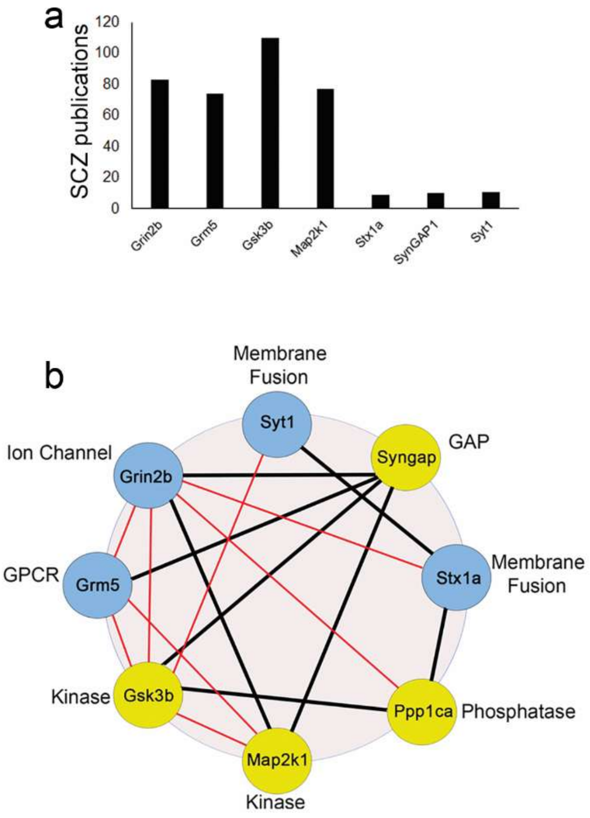

Supplementary Figure 1

Supplementary Figure 1. **a**, The number of publications in Pubmed (6/2023) using gene name of the target proteins and “schizophrenia” as search terms. MAP2K1 was searched with its alternative name, MEK1. **b**, Known interactions between the targets. Blue circles represent a transmembrane protein and yellow circles represent a soluble protein. Black lines indicate a physical protein interaction described in the BioGRID database and red lines indicate a functional interaction described in the literature. The number of proteins identified in the bait(**c**) and control(**d**) IPs

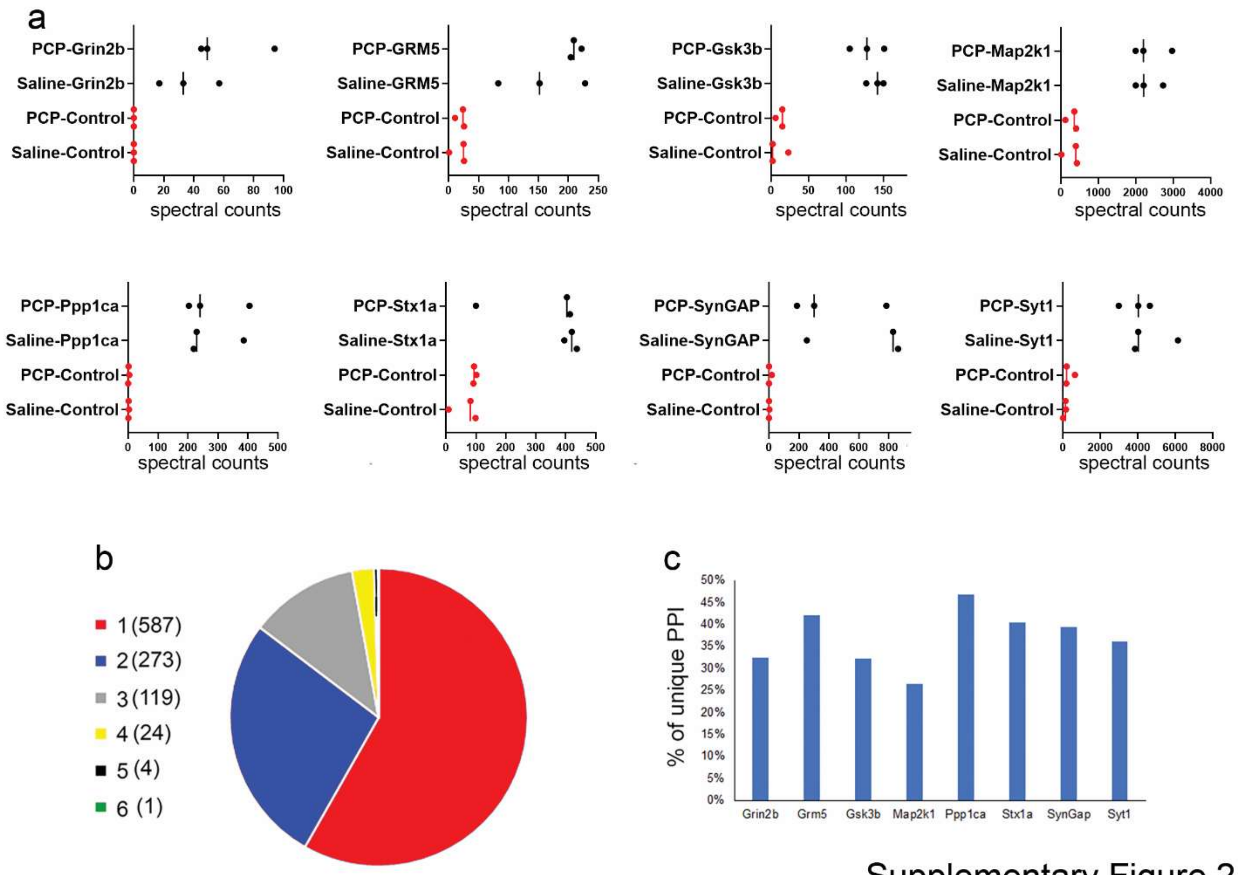

Supplementary Figure 2

Supplementary Figure 2. **a**, The abundance of the baits identified in the bait and control IPs. X-axis is spectral count. **b**, Number of baits assigned to an interactor. **c**, Percentage of unique interactors assigned to each bait network.

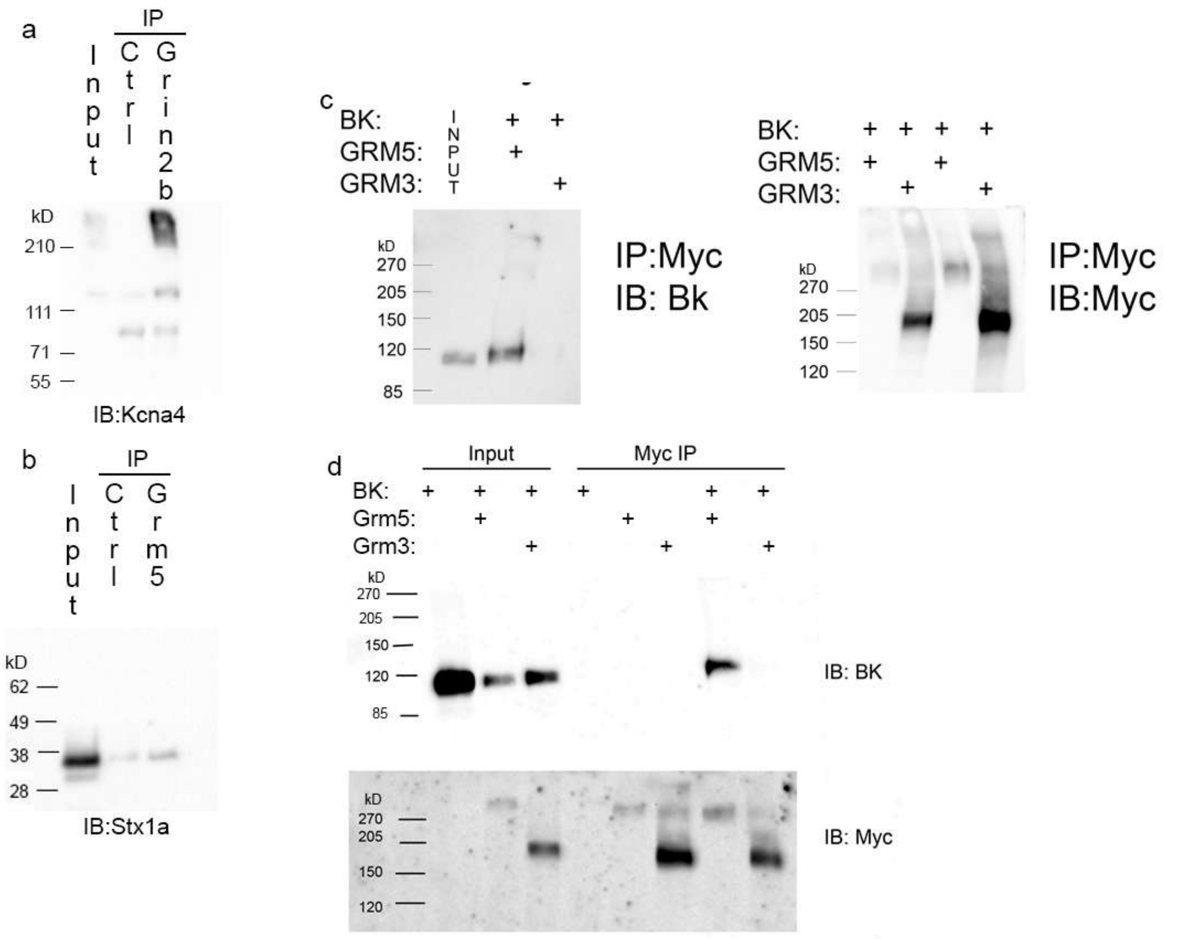

Supplementary Figure 3. a, Immunoblot analysis of Grin2b and Control (Ctrl) immunoprecipitates from rat hippocampi were probed with a Kcna4 antibody. b, Immunoblot analysis of Grm5 and Control (Ctrl) immunoprecipitates from rat hippocampi were probed with a Stx1a antibody. c, HEK cells were transfected with plasmids containing untagged Bk-alpha (i.e., Kcnma1) Grm5-myc, Grm3-myc cDNA. Lysates were immunoprecipitated with a Myc antibody and probed with a Bk-alpha (right panel) or Myc (left panel) antibody on an immunoblot. d, N2A cells were transfected with plasmids containing untagged Bk-alpha (i.e., Kcnma1), Grm5-myc, Grm3-myc cDNA. Lysates were immunoprecipitated with a Myc antibody and probed with a Bk-alpha (top panel) or Myc (bottom panel) antibody on an immunoblot.

Supplmentary Figure 4

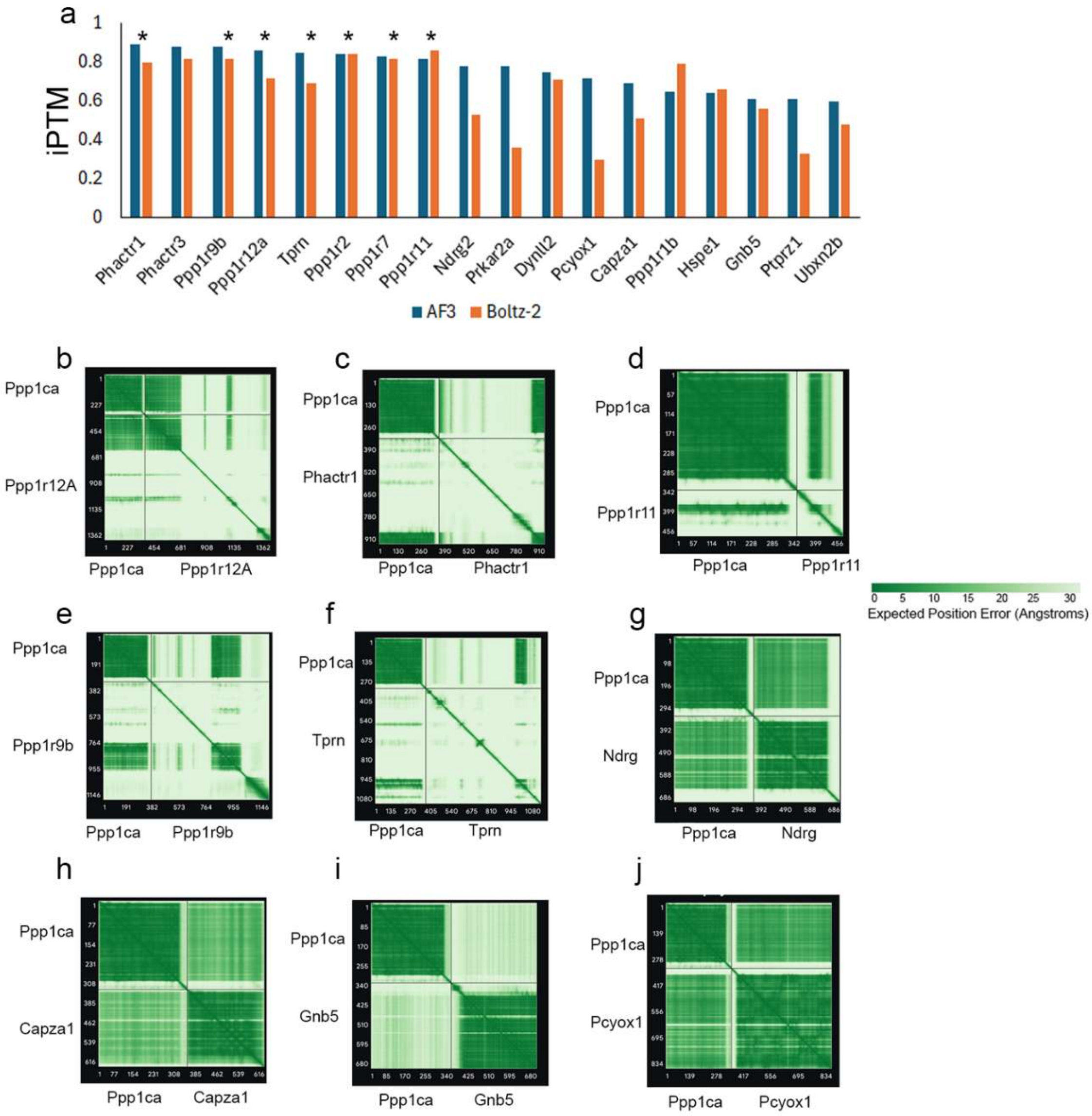

Supplementary Figure 4. a, Comparison of iPTM scores from AF3 (blue) with Boltz-2 (orange). * indicates the PPI was previously reported as a direct interactor to PPI. AF3 PAE plots for known direct interactors (b-f) and novel interactors (g-j). Green

represents the confidence of the relative orientation between two AA with the dark hue more confident. Each protein AA is plotted from the N- to the C-terminus with the AA numbered continuously with the first AA being the first residue of Ppp1ca and the last numbered AA is the last residue of the identified PPI. Ppp1ca is 330 AA, then begins the residues of the identified PPI.
